# A pilot study on fingerprinting *Leishmania* species from the Old World using Fourier transform infrared spectroscopy

**DOI:** 10.1007/s00216-017-0655-5

**Published:** 2017-10-28

**Authors:** Andrea Hornemann, Denise Sinning, Sofia Cortes, Lenea Campino, Peggy Emmer, Katrin Kuhls, Gerhard Ulm, Marcus Frohme, Burkhard Beckhoff

**Affiliations:** 1 Department 7.2 Cryophysics and Spectrometry, Physikalisch-Technische Bundesanstalt, Abbestr. 2-12, 10587 Berlin, Germany; 2 Division of Molecular Biotechnology and Functional Genomics, Technical University of Applied Sciences, Hochschulring 1, 15745 Wildau, Germany; 3 Global Health and Tropical Medicine Center (GHTM), Instituto de Higiene e Medicina Tropical (IHMT), Universidade Nova de Lisboa, Rua Junqueira 100, 1349-008 Lisbon, Portugal

**Keywords:** Fourier transform infrared spectroscopy, Hierarchical cluster analysis (HCA), Principal components analysis (PCA), *Leishmania*, DNA, Multivariate differentiation

## Abstract

*Leishmania* species are protozoan parasites and the causative agents of leishmaniasis, a vector borne disease that imposes a large health burden on individuals living mainly in tropical and subtropical regions. Different *Leishmania* species are responsible for the distinct clinical patterns, such as cutaneous, mucocutaneous, and visceral leishmaniasis, with the latter being potentially fatal if left untreated. For this reason, it is important to perform correct species identification and differentiation. Fourier transform infrared spectroscopy (FTIR) is an analytical spectroscopic technique increasingly being used as a potential tool for identification of microorganisms for diagnostic purposes. By employing mid-infrared (MIR) spectral data, it is not only possible to assess the chemical structures but also to achieve differentiation supported by multivariate statistic analysis. This work comprises a pilot study on differentiation of *Leishmania* species of the Old World (*L. major*, *L. tropica*, *L. infantum*, and *L. donovani*) as well as hybrids of distinct species by using vibrational spectroscopic fingerprints. Films of intact *Leishmania* parasites and their deoxyribonucleic acid (DNA) were characterized comparatively with respect to their biochemical nature and MIR spectral patterns. The strains’ hyperspectral datasets were multivariately examined by means of variance-based principal components analysis (PCA) and distance-based hierarchical cluster analysis (HCA). With the implementation of MIR spectral datasets we show that a phenotypic differentiation of *Leishmania* at species and intra-species level is feasible. Thus, FTIR spectroscopy can be further exploited for building up spectral databases of *Leishmania* parasites in view of high-throughput analysis of clinical specimens**.**

**Electronic supplementary material:**

The online version of this article (10.1007/s00216-017-0655-5) contains supplementary material, which is available to authorized users.

**Graphical abstract:**
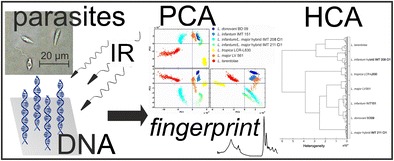
For *Leishmania* species discrimination, sample films of intact parasites and their extracted DNA were analyzed by FTIR micro-spectroscopy. Hyperspectral datasets that comprise mid-infrared fingerprints were submitted to multivariate analysis tools such as principal components analysis (PCA) and hierarchical cluster analysis (HCA).

## Introduction

Leishmaniasis is a parasitic disease caused by intracellular protozoan parasites belonging to the genus *Leishmania*, family *Trypanosomatidae* and are transmitted to mammals through the bite of female phlebotomine sand flies. These protozoa comprise approximately 53 species with an ongoing debated taxonomical structure. Currently 31 species are parasites of mammals and at least 21 *Leishmania* species are known to cause disease in humans [1, 2].

Over the past 20 years, the existence of natural hybrids is a result of genetic recombination between different *Leishmania* species such as *L. panamensis/L. braziliensis, L. braziliensis/L. peruviana*, and *L. major/L. infantum* described in [3–5]. Depending on the parasite species and the immune system of the host, the disease can cause different clinical forms ranging from localized self-limiting and self-healing cutaneous lesions – cutaneous leishmaniasis (CL) – to visceralizing infections – i.e., visceral leishmaniasis (VL), which is fatal if left untreated [2]. Depending on the *Leishmania* species, the disease can be zoonotic or anthroponotic. Frequent animal hosts are dogs and other canids, rodents, hyraxes, and marsupials and, more recently, cats [6–8]. The World Health Organization (WHO) states in its Fact Sheet [9] leishmaniasis have more than 90 different sand flies capable of transmitting *Leishmania. Leishmania* infections can also be transmitted via contaminated syringes [10] and potentially from mother to child [11].

Leishmaniasis occurs in more than 98 predominantly tropical and subtropical countries on four continents with an estimated number of new cases of 0.7–1.2 million for cutaneous leishmaniasis (CL) and 0.2–0.4 million for visceral leishmaniasis (VL) per year, with an overall prevalence estimated to be 12 million [12, 13].

Biological and genetic traits of both host species and *Leishmania* strongly determine how the disease will evolve. Thus, a correct identification of the parasite(s) is essential, as it may have implications for diagnosis, epidemiology, treatment, and control of the disease [1, 14, 15].

The most common diagnostic method for leishmaniasis is the detection of *Leishmania* amastigotes (non-flagellated intracellular forms) by microscopic observation in Giemsa stained tissue biopsies of infected patients. Additional evidence of an infection is the presence of *Leishmania* parasites in cultures inoculated with suspected biological tissue samples. However, these methods are not always successful and lack differentiation capacity, as *Leishmania* species cannot be distinguished morphologically. In the 1980s, isoenzyme analysis, also called multilocus enzyme electrophoresis (MLEE), became the ‘gold standard’ for typing *Leishmania* at the species and intra-species levels [16–19]. MLEE needs cultured parasites, which is labor-intensive, time-consuming, and can only be performed in specialized laboratories. Moreover, it has limitations concerning discriminatory power [14, 20]. Currently this technique is being complemented and will likely be replaced in the future by molecular approaches. These approaches are based on the detection of parasitic DNA in clinical material or from cultured parasites amplified by polymerase chain reaction (PCR). Fragment size or sequence analysis of the PCR amplicons enables further characterization as well as species and strains discrimination. Most commonly used methods are restriction fragment length polymorphism (PCR-RFLP) analysis and sequencing of single markers or multilocus sequence typing (MLST) as well as multilocus microsatellite typing (MLMT) [1, 13, 20, 21].

In developing countries, where usually simple and inexpensive techniques are required, the need for trained personnel and well equipped laboratories still comprises a huge obstacle. Thus, cost-effective and simple methods for the early-stage diagnosis and parasite identification are needed [22–24]. To overcome these obstacles, optical methods such as vibrational spectroscopy using infrared radiation in the mid-infrared (MIR) spectral range can be implemented. For rapid and accurate identification as well as discrimination of microorganisms at the genus, species, and strain level, only small sample amounts without any complex manipulation are required [25, 26]. Fourier transform infrared spectroscopy (FTIR) instrumentation is available in highly equipped laboratories; however, the sample preparation can also take place in low resource settings with an easy transfer of the substrate slides to the specialized ones. FTIR spectroscopy allows the sensitive and noninvasive analysis of IR light interaction with a molecule, and hence functional groups can be determined by absorption, emission, or reflection profiles.

**Fig. 1:**
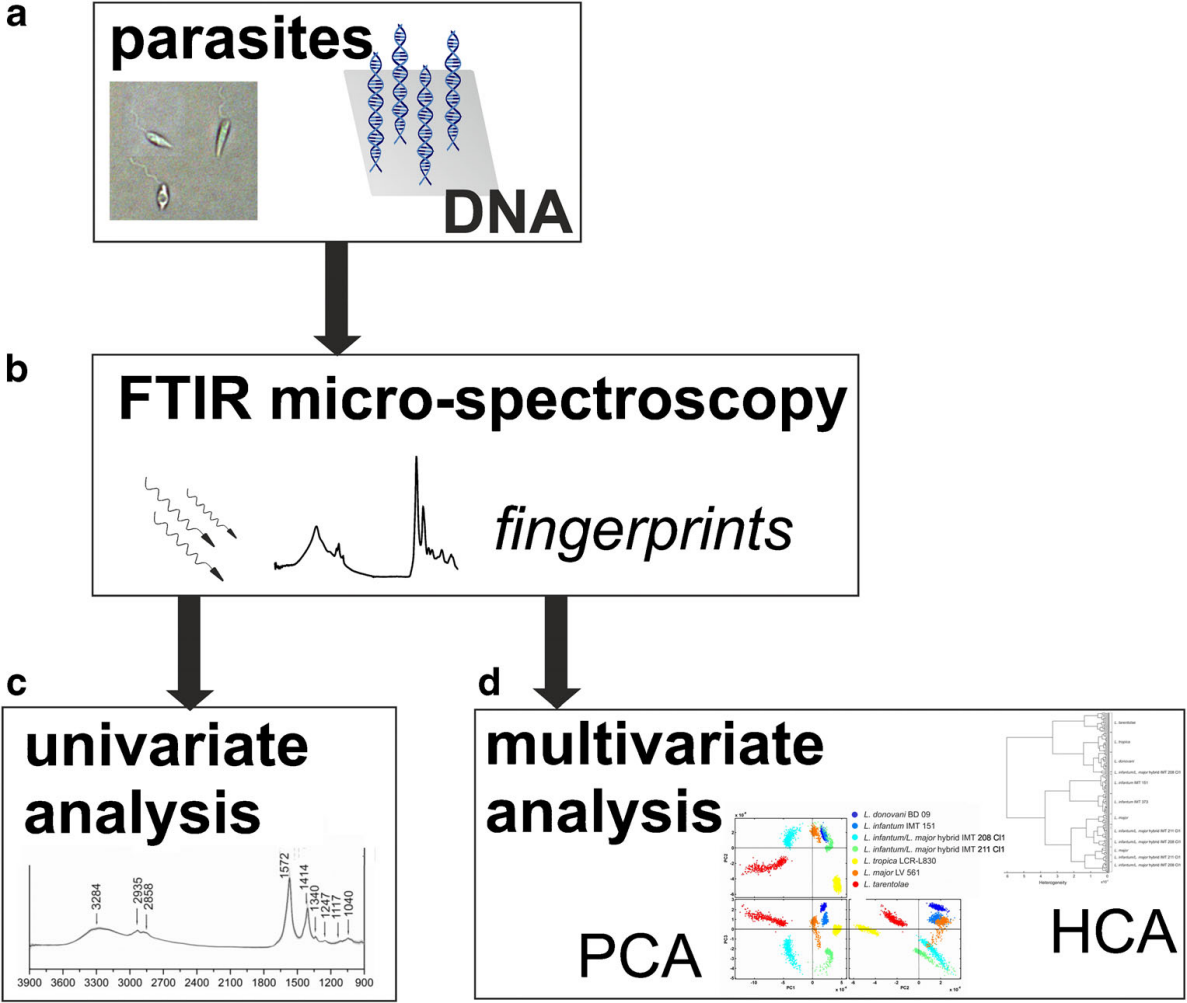
Scheme of the MIR strategy towards *Leishmania* species discrimination (identification, typing): sample films of intact parasites and their extracted DNA as representatives for the *Leishmania* species **(a)** were analyzed by FTIR micro-spectroscopy for the acquisition of hyperspectral datasets that comprise MIR spectral fingerprints enabling identification **(b)**. Univariate analysis allows the chemical composition to be studied, enabling discrimination by characteristic modes and their intensity variations **(c)**. Multivariate analysis tools enable variance-weighted cross-correlation of MIR fingerprints (PCA) and distance-based species differentiation (HCA) for studying the multi-dimensional characteristics **(d)**

This methodology in combination with various multivariate statistic analysis tools could be successfully implemented for distinct identification and differentiation of biological microorganisms such as bacteria [27, 28], e.g., for taxonomic differentiation of *Lactobacilli* [29] or *Streptomyces* [30]. FTIR supported by artificial neural network (ANN) analysis has shown its potential for accurate discrimination of *Listeria* strains [31]. FTIR is an easily accessible, label-free, and potentially powerful tool for studies on *Leishmania* parasites. The data acquisition in the MIR window reflects the overall composition that often differs with respect to their molecular make-up, thus providing unique fingerprint signatures for differentiation. An initial approach to infrared (IR) data-based differentiation of parasite sample films of three *Leishmania* species has been conducted successfully by Aguiar et al. [32]. Further, studies on malaria parasites have been reported [33]. However, as there is an insufficient number of IR-based approaches on identification of protozoan parasites, further studies are needed in order to elucidate their biochemical diversity under the implementation of computerized chemometric tools. Our systematic pilot study in the MIR spectral region from 3900 to 400 cm^–1^ is the first step to achieving this ultimate goal by unraveling the molecular composition and complexity of the selected *Leishmania* strains, the hyperspectral datasets of which were analyzed by supervised and unsupervised multivariate statistics tools, namely principal components analysis (PCA) and hierarchical cluster analysis (HCA). A scheme on this approach is illustrated in Fig. 1 *.*

The aim of this work is to use several *Leishmania* species and species-hybrids, such as *L. infantum*, *L. major*, *L. tropica*, *L. donovani*, *L. tarentolae*, *L. infantum/L. major* hybrids, and their molecular building blocks, such as their DNA, to assess the efficiency of FTIR in terms of reliable and comparative high-throughput analysis, especially in view of diagnosis. In association with chemometric tools [34], it is shown that FTIR methodology accurately discriminates structural patterns of spectral datasets and elicits a specific hierarchy that may help to achieve a better understanding of complex biological systems diversity and the associated biochemical nature.

The applicability of FTIR spectroscopy as a routinely implementable bioanalytical tool based on robust and reproducible MIR spectral features of various *Leishmania* samples will be presented. Our pilot study focuses on the evaluation of MIR fingerprints in order to build up spectral databases enabling identification of the causative species/strains of *Leishmania* infections in the near future.

## Materials and methods

### *Leishmania* strains

Several strains and clones of the humanpathogenic species *Leishmania infantum*, *L. major*, *L. donovani*, *L. tropica*, and *L. infantum/L. major* hybrids were included in this study, in addition the non-pathogenic species *L. tarentolae* (Table 1). The hybrid *L. infantum*/*L. major* strains were reported for the first time by Ravel et al. in Portugal [5].

**Table 1:** *Leishmania* species and hybrids and respective strains used in the present study

Species	Laboratory code	WHO code	Pathology	(No.^a^, No.^b^)
*L. donovani*	BD 09 BD 12 BD 14 BD 17	MHOM/BD/2006/BD09 MHOM/BD/2006/BD12 MHOM/BD/2006/BD14 MHOM/BD/2006/BD17	VL VL VL VL	(3,2) (2,1) (2,1) (2,1)
*L. infantum*	IMT 151 IMT 151 Cl1 IMT 151 Cl2 IMT 373	MHOM/PT/1988/IMT151 MHOM/PT/1988/IMT151cl1 MHOM/PT/1988/IMT151cl2 MCAN/PT/2005/IMT373	VL VL^c^ VL^c^ CanL	(3,2) (2,1) (2,1) (2,1)
*L. major*	LV 561 LV 561 Cl1 LV 561 Cl2 LV 561 Cl3	MHOM/IL/1967/LRC-L137 JerichoII MHOM/IL/1967/LRC-L137 JerichoIIcl1 MHOM/IL/1967/LRC-L137 JerichoIIcl2 MHOM/IL/1967/LRC-L137 JerichoIIcl3	CL CL^c^ CL^c^ CL^c^	(3,2) (2,1) (2,1) (2,1)
*L. infantum/L. major* hybrids	IMT 208 Cl1	MHOM/PT/1994/IMT208cl1	VL^c^	(3,2)
*L. infantum/L. major* hybrids	IMT 211 Cl1	MHOM/PT/1994/IMT211cl1	VL^c^	(2,1)
*L. tropica*	LCR-L830 LCR-L1322 LCR-L881	MHOM/PS/2001/ISL593 MHOM/PS/2008/335jnM59 MHOM/PS/2002/63jnF21	CL CL CL	(2,1) (2,1) (2,1)
*L. tarentolae* ^d^		n.a.	non human pathogenic	(3,2)

VL, visceral leishmaniasis; CanL, canine leishmaniasis; CL, cutaneous leishmaniasis; n.a., not available;

^a^Number of parasitic film preparations

^b^Number of DNA film preparations

^c^Pathology such as for the uncloned strain

^d^Outgroup (*Sauroleishmania*)

### Sample preparation

#### Cultivation of *Leishmania* strains

*Leishmania* strains were maintained in cell culture flasks (Sarstedt) with M199 medium (Sigma Aldrich) supplemented with 2.2 g/L NaHCO_3_, 10% fetal calf serum (BioChrom AG), 1% L-glutamine, and 0.5% penicillin/streptomycin (Sigma Aldrich). A neutral pH was ensured by the addition of 1 M HEPES-NaOH buffer solution (pH 6.9). The cultures were kept in an incubator at 26 °C and every 3 to 4 d fresh medium was added by diluting the cultures 1:5 to 1:10. The density of promastigotes (flagellated stage of the parasites) was determined by microscopy using a Neubauer improved cell counting chamber (VWR).

#### Isolation of *Leishmania* DNA

A volume of 3–12 mL parasite culture with a density of approximately 10^6^ parasites/mL was used for DNA extraction. After centrifugation (3000 rpm) for 8 min, the supernatant was discarded and the remaining pellet was washed twice with 1 mL ultrapure water (18.2 MΩ∙cm, Merck KGaA), and centrifuged at 3000 rpm for 8 min. Then, the pellet was resuspended in ultrapure water and centrifuged at 3000 rpm for 8 min. The purified pellet was redissolved in 1 mL lysis buffer (50 mM NaCl, 10 mM EDTA, and 50 mM Tris-HCl, pH 7.4), followed by the addition of SDS to a final concentration of 0.5% and proteinase K (20 mg/mL) to a final concentration of 100 μg/mL and transferred to an Eppendorf tube.

The batch for cell lysis was incubated over night at 55 °C with moderate shaking (300 rpm) in a thermo-mixer (Thermomixer Comfort, Eppendorf). An equal volume of phenol/ chloroform/isoamyl alcohol (25:24:1 v/v/v) was added and the tube was gently shaked for 2 to 3 min. Afterwards, tubes were centrifuged at 16,000 × *g* for 10 min and the aqueous phase was transferred into a new tube. This extraction step was repeated two times. Finally, an equal volume of chloroform-isoamyl alcohol (24:1, v/v) was added, gently mixed, and centrifuged as previously. The aqueous phase was removed carefully to a new tube and 1/10 volume of 3 M sodium acetate and an equal volume of isopropanol were added for DNA precipitation. After mixing gently, the tubes were kept overnight at –20 °C. After centrifugation at 16,000 × *g* for 30 min, the supernatant was carefully discarded and the DNA pellets were thoroughly washed twice by addition of 0.5 mL 70% ice-cold ethanol and centrifugation at 16,000 × *g* for 15 min. The reaction tubes were kept open for complete ethanol evaporation. Finally, DNA was dissolved in 15–25 μL of ultrapure water for several hours in a thermo-mixer at 42 °C and at 300 rpm. DNA concentrations and quality were determined with a spectrophotometer (NanoDropTM 1000; Thermo Scientific). Details can be found in Fig. S1 in the Electronic Supplementary Material (ESM).

#### Sample preparation of parasites and DNA films for FTIR analyses

Critical steps that may have impact on the yield were harvesting and washing *Leishmania* parasites from cultures, application onto the optical window, and thorough drying of the intact parasite samples into films. Another critical issue is the different growth kinetics of *Leishmania* promastigotes in culture, as variations in growth rate may result in unequal final concentrations of cells. Therefore, all samples were collected between the 4^th^ and 5^th^ d in culture to ensure a constant concentration of the parasites. Promastigote cultures of each strain were adjusted to equal concentrations for application onto the FTIR windows and to avoid large film thickness variations during FTIR assessments. However, small variations in film thickness could be mitigated by statistical considerations (i.e., by applying standard deviation, arithmetic mean, and multivariate methodologies). A volume of 0.5–1 mL of culture medium containing a defined density of 10^6^ parasites/mL was considered to be the minimum sample amount needed for reliably conducting infrared spectroscopical measurements. These parasite culture suspensions were centrifuged at 1000 × *g* for 8 min. The supernatant was eliminated thoroughly and the respective pellet was washed three times with centrifugation at 1000 × *g* for 8 min with a saline solution (0.9 % NaCl) and, finally the pellet was resuspended in 20 μL of ultrapure water.

From this batch, about 2 μL droplets were pipetted (three times in total at the same place) onto reflective MirrIR low-emissivity (“low-e”) microscope slides (Kevley Technologies) and air-dried, in order to guarantee a dense homogeneous sample film for FTIR spectroscopic analysis.

In the same way, several sample droplets of the respective isolated DNA were prepared onto low-e-slides utilizing a hotplate for drying at 30 °C.

## FTIR spectroscopy – experimental setup and data acquisition

### Micro-spectroscopic studie**s**

Absorbance spectra of intact *Leishmania* films and their related DNA were recorded in reflection geometry in the MIR spectral range between 3900 cm^–1^ and 900 cm^–1^. This was done using a Vertex 80v FTIR spectrometer (Bruker Optics GmbH) to which a FTIR Hyperion 3000 microscope was coupled. The spectrometer was fitted with a KBr beamsplitter and a globar was implemented as a radiation source for micro-spectroscopic investigations.

For data acquisition, a lN_2_-cooled multi-element mercury cadmium telluride detector, a so-called focal plane array (FPA) detector with 128^2^ pixel elements and a spectral resolution of 4 cm^–1^ was used. Micro-spectroscopic experiments on sample films were conducted with a Cassegrain objective at a 15× magnification, enabling the study of a sample area of 345^2^ μm^2^ at approximately 2.87 μm lateral resolution; the latter corresponds to the dimension of one single pixel.

Each spectrum was collected with the Opus software v.7.2 (Bruker Optics GmbH) and consisted of 512 averaged scans for parasite sample films. For DNA films, 128 averaged scans were accumulated. All interferogram scans were submitted to a Blackman Harris 3-term window function and to a zero-filling factor of 2 prior to Fourier transformation. Background scans were collected prior to each sample measurement from a region free of samples, here on a clean low-e-slide, and rationed against the sample spectrum.

For the approval of instrumental invariance the *Leishmania* strains were measured in duplicate. For the DNA measurements, one to two spectral datasets comprising 480 spectra, respectively, were analyzed (Figs. S2 and S3, ESM). Despite a low number of DNA replicates the datasets display a similar mutual statistical variability being reflected by boxplots (Fig. S3, ESM). These boxplots (highlighted in green, blue, and red) correspond to the number of sample film preparations (Table 1). The strains *L. infantum* IMT 151, *L. donovani* BD 09, the hybrids *L. infantum/L. major* IMT208 Cl1 and IMT211 Cl1, and *L. tarentolae* were prepared one further time and measured again, finally resulting in three sample preparations for these strains.

Duplicate and, when possible, triplicate preparations of the *Leishmania* species were conducted to verify that preparation steps for the cultivation of the *Leishmania* species and PCR-based procedures were reliable and consistent.

### Univariate- and multivariate data analysis

The FTIR micro-spectroscopic datasets were subjected to the imaging software CytoSpec v.1.4.03 and cut to the 3900–900 cm^–1^ spectral range. Afterwards, a baseline correction (polynomial fit procedure of 3^rd^ order, 6–7 correction points) of areas that comprised 420 spectra per sample system in the case of the parasite film data, and 480 spectra per sample system in the case of the DNA films, were further analyzed. The different number of scans for parasite and DNA sample systems was taken to ensure optimal peak-to-noise ratios. We also paid attention to recording spectra in regions with approximately the same thickness, which can be explained by the different number of spectra taken from the parasites and DNA sample films datasets. Furthermore, this optimization equally included a scan number adaptation to the sample mass depositions of the sample areas which comprised the functional groups of interest.

The data processing software Origin 9.0G was implemented for analysis on spectral averages and their corresponding standard deviations. Normalization of the spectral datasets between 0 and 1 was performed for the sake of a better comparison in the spectral range 3900–900 cm^–1^. For the determination of prominent bands, peak analysis (with a threshold height of 5%–10%) on spectral averages of the respective *Leishmania* strain was performed in Origin 9.0G, as well as the construction of boxplots. For boxplot construction, 100 normalized spectra for the respective sample system were considered (ESM, Figs. S2 and S3). The selection of these 100 spectra was a random choice to get a full picture of their statistical distribution. We utilized the boxplots to display the variation and statistical distribution of the MIR datasets, which can be considered as a statistical population. The boxplots are divided into five points, the median, two quartiles, and the minimum and maximum of all the data. The position of the median provides information about the existence of the symmetry or skewness of a distribution.

For the multivariate analysis, the software Matlab R2012a and Toolboxes Stats Toolbox (Mathworks) and PLS Toolbox (Eigenvector Research Inc.) were used. Multivariate analyses were performed in diverse spectral windows in two different ways:

(1) Principal components analysis (PCA) was performed by applying data pretreatments such as vector-normalization, mean-centering, and 2^nd^ derivatives with the help of the Savitzky-Golay-algorithm and five smoothing points. PCA was carried out in diverse spectral windows and in combinations of the latter for elucidating the highest differentiation capability among the respective parasites and DNA datasets (Table 2). The loadings spectra and scores were calculated in Matlab R2012a. The spectral loadings were plotted in Origin 9.0G.

**Table 2:** Spectral windows selected for PCA

Sample films	Wavenumber window/cm^–1^	No.
Intact parasites	3000 – 2700 1800 – 1500 1500 – 1200 1200 – 900	W1 W2 W3 W4
DNA	3000 – 2700 1750 – 1450 1450 – 1250 1250 – 900	A B C D

(2) Agglomerate hierarchical cluster analysis (HCA) was performed by applying the Euclidean distance measure and Ward’s algorithm. For HCA, 20 datasets of the score matrix per sample were used. Second derivative IR spectra to calculate spectral distances were found to be useful for hierarchical clustering.

## Results and discussion

### Univariate studies – chemical analyses on intact parasite films and DNA

#### Reproducibility tests

The main critical step for reliable spectroscopic analysis on *Leishmania* parasite films entailed the thorough elimination of the culture medium. Therefore, several washing steps were performed, as remaining additives may cause competitive spectral contributions. For instance, vibrational spectroscopic features from fetal calf serum, as one of the main components in the culture medium, may coincide with parasitic proteinogenic amide modes.

To evaluate the reliability and robustness of FTIR spectral datasets of the six strains, *L. infantum* IMT 151, *L. infantum/ L. major* hybrids IMT 208 Cl1 and IMT 211 Cl1, *L. donovani* BD 09, *L. tropica* LCR-L881, and *L. tarentolae* were considered. As FTIR data must show a Gaussian distribution for submitting them to multivariate statistics, their consistency was verified and approved with the help of boxplots of the respective sample film preparations (Figs. S2 and S3, ESM).

Furthermore, the resulting spectra of remeasurements (i.e., a second measurement of the same sample films but in another region of interest) were compared with the spectra from the previous acquisition using PCA and were found to be very similar, that is, clustering closely together and indicating proper reproducibility of the instrumental setup (data not shown). In addition, boxplot analyses (Figs. S2 and S3, ESM) display high similarities within the film preparations of the respective parasites and their DNA (this was tested with all 18 strains and 2–3 sample preparations per strain), and reproducibility of their spectral datasets could be successfully envisaged.

#### *Leishmania* parasite films

**Fig. 2:**
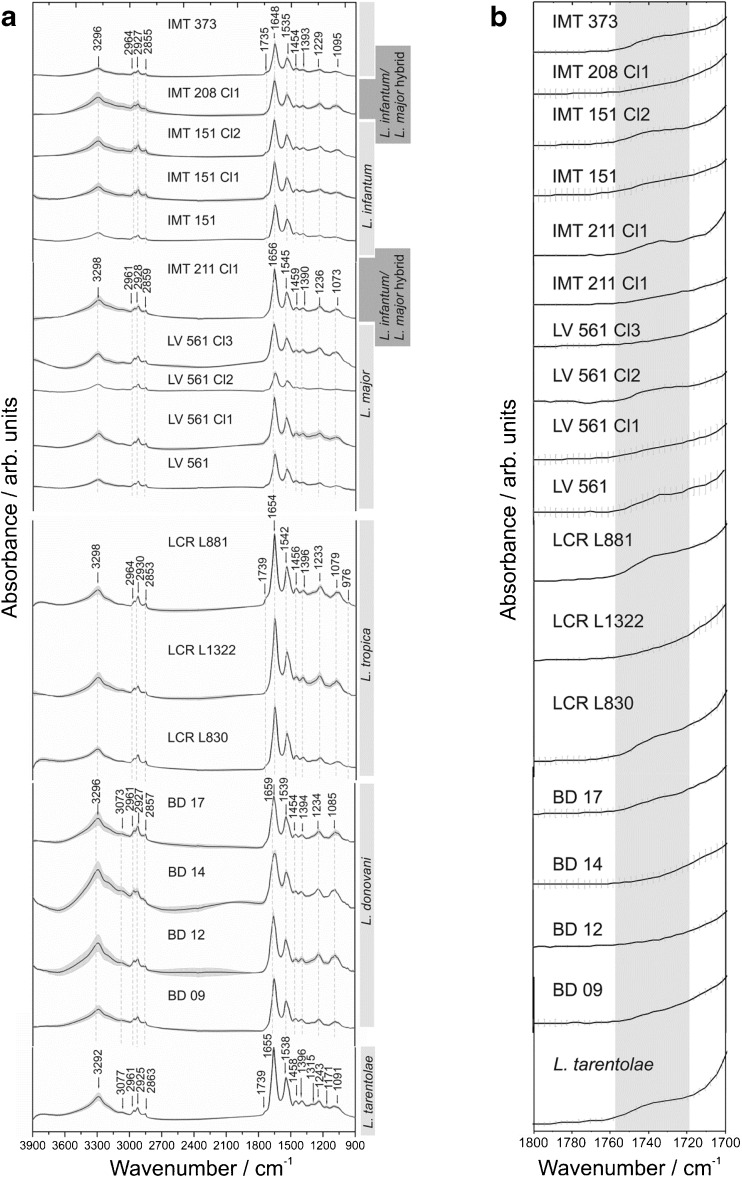
**(a)** MIR spectral fingerprints of the *Leishmania* parasites’ films from cultures of the 18 studied strains with their arithmetic means (each spectral fingerprint is the arithmetic mean of 420 spectra) and standard deviations (gray envelopes), respectively. Analysis was performed in the 3900–900 cm^–1^ spectral region at 4 cm^–1^ spectral resolution. **(b)** Region of the C=O stretching vibration which appears as an additional shoulder of the Amide I mode for some of the *Leishmania* datasets (IMT 373, IMT151 Cl2, IMT 211 Cl1, LV 561 Cl2, LV 561, LCR L881, LCR L830, *L. tarentolae*)

Spectral datasets with their standard deviations (represented in gray envelopes, Fig. 2) of the parasite films of all 18 strains are listed in Table 1. Included therein are different species as well as different strains of species resembling the position of bands and forms typical of IR spectral datasets of whole microorganisms such as bacteria [35, 36].

Also for diagnostics, dried films of body fluids, serum proteins, or other fluid specimens are convenient for routine FTIR-based probing [37, 38]. Subsequently, the difference in several bands related to the corresponding functional groups will be discussed for the sample films of the respective parasite species.

FTIR spectra obtained from *Leishmania* parasite samples exhibited characteristic molecular fingerprints, and the replicates indicated good reproducibility for each sample preparation (Figs. S2 and S3, ESM).

Figure 2 illustrates the calculated arithmetic means of 420 spectra for the respective sample hyperspectral dataset, which comprise a huge number of approximately 6,532,000 data points in total. IR datasets display a high similarity among all parasitic strains. Previous investigations on three very similar IR datasets of *L. amazonensis*, *L. chagasi* (or *L. infantum*), and *L. major* parasitic strains [32] have shown spectral differences between these species allowing species discrimination/typing in the regions of polysaccharides, fatty acids (phospholipids), nucleic acids, and proteins (amides). Indeed, modes from molecular constituents such as polysaccharides, nucleic acids, amino acids, lipids, and proteins can be detected for the *Leishmania* sample film datasets. Similar to what was observed for other microorganisms such as bacteria in the MIR spectral window [39]. We also noticed spectral differences for *L. infantum* IMT 373, *L. tropica* LCR L881, and *L. tarentolae* in comparison with the remaining strains in these depicted wavenumber regions. For instance, we can observe the presence/absence of a shoulder at about 1739 cm^–1^ originating from the amide II mode region (Table 3) for all investigated strains. Table 3 highlights main bands observed in the *Leishmania* spectra in the respective wavenumber regions, together with associated functional group modes, which can be expected from microorganisms according to Helm et al. [27]. The fatty acid region (3000–2800 cm^–1^) comprises CH_3_, CH_2_, and CH stretching vibrations of functional groups that can be found in cellular membranes. In the 1800–1500 cm^–1^ spectral range, amide bonds can be identified, which include vibrations of carboxyl, carbonyl, and ketone groups of various proteins and peptides. In this spectral region, amide I and amide II bands can be found at about 1650 cm^–1^ and 1550 cm^–1^, respectively [40]. Bands of high intensity occur in the wavenumber window 1580 cm^–1^–1465 cm^–1^. The so-called ‘mixed region’, in which vibrations of proteins, lipidic acids, and phosphate compounds can be identified, is located at wavenumbers 1500 cm^–1^–1200 cm^–1^. Modes in the spectral region between 1200 cm^–1^ and 900 cm^–1^ refer to vibrations of polysaccharides originating from the membrane surface of *Leishmania* parasites (Table 3). These band intensities differ slightly among the species complexes (i.e., *L. major* with *L. tropica*) and within one species (*L. major* LV561 Cl2 with *L. major* LV561 Cl3).

**Table 3:** Band assignments that can be found in IR spectra of *Leishmania* parasites’ films at the wavenumber regions according to Helm *et al.* [27, 41], and observed bands in this study

Observed bands / cm^-1^	Wavenumber region(s) [27, 41] / cm^-1^	Designation	Band assignments
3296, 3296, 3292	3300	Intermol. bonded, water, carboxylic acid	OH, NH from amines
3220, 3135, 3000, 2930, 2885, 2850, 2798, 2779	3000–2800	Fatty acid region	CH_3_, CH_2_ and CH stretching vibrations of fatty acid residues, phospholipids
1739,1660, 1580	1800–1500	Amide region	carboxyl, carbonyl, and keto groups of proteins and peptides; contains amide I and II bands
1465, 1380, 1315, 1220	1500–1200	Mixed region	proteins, fatty acids and phosphate compounds,
1095–1073 1160, 1100, 1009, 985	1200–900	Polysaccharide region	phosphodiester bonds (from DNA) polysaccharides in surface membrane

The spectral fingerprints comprise vibrational modes assigned to compounds that originate from different cell organelles. Aside from typical eukaryotic organelles such as the Golgi apparatus, endoplasmatic reticulum, and mitochondria, *Leishmania* possess a flagellum (promastigote culture forms) and a kinetoplast that comprise DNA in the form of maxi- and minicircles [42]. The plasma membrane of *Leishmania* is comprised of a glycocalyx, which entails glycoconjugates that are anchored to the plasma membrane via glycosylphosphatidylinositol (GPI). The glycoconjugates are glycoproteins, in particular proteophosphoglycan (PPG) and zinc-metalloprotease GP63, also called leishmanolysin and glycolipids, whereas lipophosphoglycan (LPG) is the most abundant [42, 43]. The LPG of *Leishmania* promastigotes play key role in the parasite’s survival in both the insect host being responsible for the docking to the sand fly intestine or also in mammalian hosts, by decreasing phagosome fusion properties at the onset of infection in macrophages [44, 45]. PPG, on the other hand, is known to protect *Leishmania* parasites from hydrolases during the sand fly’s blood meal [43]. Leishmanolysin GP63 also prevents complement-mediated lysis and plays a key role with respect to the virulence of *Leishmania* parasites [42, 46]. Glycosylation of membrane components results in vibrations mainly occurring in the polysaccharide region. The phosphate compounds in the ‘mixed region’ are derived from phospholipids of the plasma membrane and DNA and RNA molecules.

In all spectra, deviations of the modes in the 3220 cm^–1^ and 1660 cm^–1^ spectral range, with respect to the presence/absence of bands and differences in band intensities can be observed. The mode at 1660 cm^–1^ displays a neighbored shoulder of the amide I band. Basically, this mode, which is located between 1710 and 1750 cm^–1^, can be assigned either to C=O vibrations from esters, which may occur in lipids or to C=O stretching modes originating from proteins. In this case, and as it is expected from micro-organisms usually containing more protein than lipid-related components, we assign this shoulder to the Amide I mode, which actually entails C=O stretching vibrations from amino acid side-chain contributions (Table 3) [47]. As bonds to atoms with a strong electronegativity of the ester group may cause a band shift [35], different molecules that can be found in *Leishmania* may consequently cause a varyingly strong peculiarity of this mode. The region at 3220 cm^–1^ refers to stretching vibrations of adsorbed water molecules and NH residues.

These findings may be a first indication for the feasibility of using reflective FTIR micro-spectroscopy to study diverse sample films of parasitic cultures with respect to their discrimination capability, especially in the spectral region of polysaccharides, fatty acids (phospholipids), nucleic acids, and proteins (amides).

#### *Leishmania* DNA films

The DNA profile of each single *Leishmania* strain can be considered a molecular fingerprint that reflects the parasite’s evolution. It is characteristic to a large extent, and can be exploited for classification of parasites by genotyping. This is an important aspect, as some of the *Leishmania* species are known to be clinically pleomorphic [15]. For this reason, we also conducted a comparative IR spectroscopic analysis on DNA films.

**Fig. 3:**
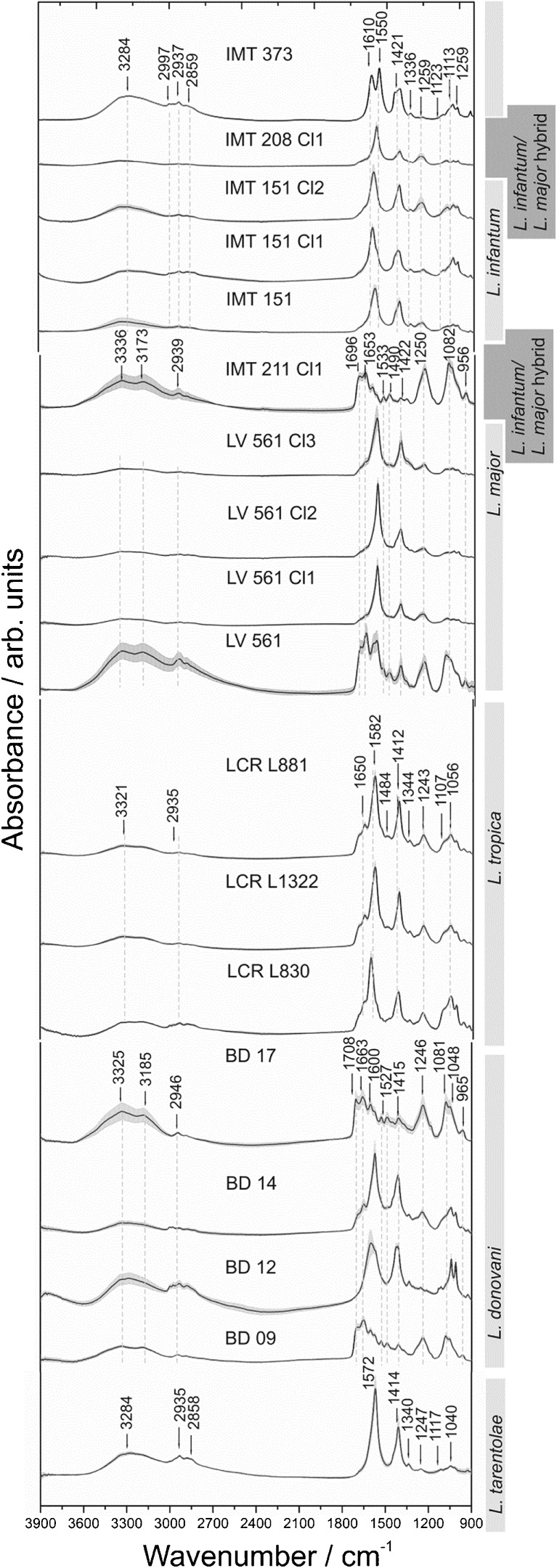
MIR spectral fingerprints including arithmetic means (each spectral fingerprint is the arithmetic mean of 480 spectra) and standard deviations (gray envelopes) of the *Leishmania* DNA films of the 18 studied strains. Analysis was performed in the 3900–900 cm^–1^ spectral region at 4 cm^–1^ spectral resolution

Figure 3 displays the calculated arithmetic means of 480 spectra per sample hyperspectral dataset, which comprises in total about 7,464,000 data-points. The DNA spectra of the different *Leishmania* strains contain typical modes at 2925 cm^–1^ CH and at 1043 cm^–1^ , where the C-O-stretching vibrations of the ribose group (Table 4) can be observed [48]. The different type and/or amounts of bases for the respective *Leishmania* strain also result in different intensities of infrared absorption. The broad spectral region between 3500 cm^–1^ and 3000 cm^–1^ can be attributed to stretching vibrations of water and NH molecules [49]. Furthermore, the DNA data show modes at 1650–1610 cm^–1^ and at about 1500 cm^–1^ that can be assigned to in-plane vibrations of cytosine, and stretching vibrations of the thymine ring at 1575 cm^–1^ (Table 4) [48, 49]. The IMT 151 and IMT 208 datasets are mutually very similar among themselves, apart from the signatures of IMT 373, IMT 211 Cl 1, LV561, BD 17, and BD 09, the latter of which comprise further modes in the spectral range between 1550 cm^–1^ and 1708 cm^–1^. Moreover, modes in the spectral range between 1550 cm^–1^ and 1300 cm^–1^ can be assigned to in-plane vibrations of residues of DNA bases and out-of-plane vibrations (800 cm^–1^–760 cm^–1^). Modes at approximately 1225 cm^–1^ and 1090 cm^–1^ refer to the antisymmetric and symmetric PO_2_^-^ stretching vibrations, which are more pronounced in some DNA spectra (Fig. 3) than in those of parasite films (Fig. 2).

**Table 4:** Band assignments that can be found in IR spectra of *Leishmania* DNA films

Observed bands/cm^–1^	Molecule residues	Band assignments [48, 49]
3336–3 173	OH, NH	Intermol. bonded, water, carboxylic acid, NH from amines
2925	CH	Weak, stretching vibration of ribose group
1708–1696	C=O	Stretching vibration from carboxylic acid
1685	NH_2_	Shoulder, scissoring vibration in cytosine
1650, 1610	Cytosine ring	Strong, *in-plane* vibration
1640	H_2_O	Strong and broad, scissoring vibration of adsorbed water molecules
1605	NH_2_, C=N	Strong, bending and stretching vibration in adenine
1580	Cytosine ring	Weak, *in-plane* vibration
1575	Thymine ring	Weak, stretching vibration
1573	NH_2_, C=N	Weak, bendingand stretching vibration in adenine
1500	Cytosine	Medium, *in-plane* vibrations
1550–1300	Base residues, NH-, CH-	Weak, *in-plane* vibrations
1225	PO_2_^-^	Strong, antisymmetric stretching vibration
1090	PO_2_^-^	Strong, symmetric stretching vibration
1043	C-O	Strong, stretching vibration of deoxyribose
925	C-C	Strong, stretching vibration; similar to band of diethyl phosphate anion
800–600	DNA bases	Weak, *out-of-plane* vibrations
1000–700	P-O, C-O NH	Medium to weak, stretching vibration Medium to weak, *out-of-plane* bending

### Differentiation of intact *Leishmania* parasites and their DNA by PCA

Based on the preliminary univariate results, this study focusses now on multivariate differentiation of *Leishmania* strains for which PCA was implemented. As vector-normalization to all datasets was conducted, the intensities in all spectral data were coherently scaled to 1. So, only spectral differences in the respective selected wavenumber windows (Table 2) were considered for multivariate analyses. These comprised the highest discrimination capability, the latter of which is reflected by the PC1 explained variances (Table S1, ESM). Here, we tested the differentiation capability of different *Leishmania* species, as well as different strains within a species, (i.e., the inter- and intra-species variability).

**Fig. 4:**
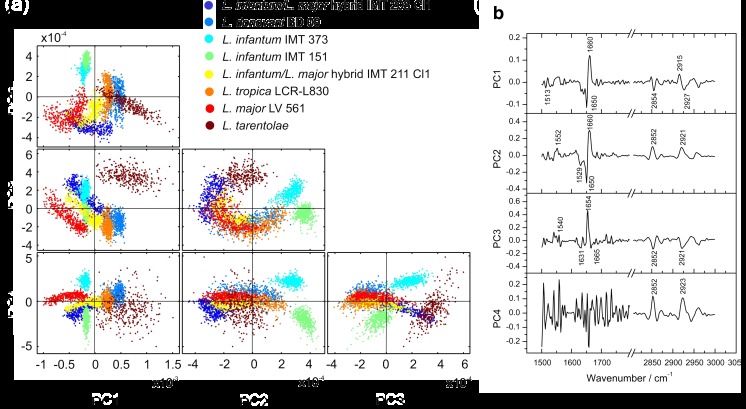
**(a)** Scores diagram (*scatter plots*) of the PCA on parasite films from PC1 to PC4. Analysis was performed applying 2^nd^ derivatives in the 3000–2700 cm^–1^ and 1800–1500 cm^–1^ spectral range. Datasets comprised 420 spectra for the strains *L. major/L. infantum* hybrid IMT208 Cl1, *L. donovani* BD09, *L. infantum* IMT151, *L. infantum* IMT373, *L. major/L. infantum* hybrid IMT211 Cl1, *L. tropica* LCR-L830, *L. major* LV561, and *L. tarentolae,* respectively. **(b)** Corresponding loadings spectra

For chemometric analysis of *Leishmania* parasite film data, one representative strain of *L. major* (LV 561), *L. tropica* (LCR-L830), *L. donovani* (BD09), *L. tarentolae* , two strains of *L. infantum* (IMT 373, IMT 151), and two *L. infantum/ L. major* hybrid strains (IMT 208 cl1, IMT 211 cl1) were selected in order to check differentiation power. This resulted in eight principal components (PCs) for the eight sample datasets. Under consideration of the wavenumber windows (Table 2) W1 (3000–2700 cm^–1^) and W2 (1800–1500 cm^–1^), the variance that can be explained for the first PC is 46.52% and the corresponding total variance captured for this PC is 83.87%. With respect to the output of the score diagrams, this combination of wavenumber windows exhibits the best results for the differentiation of *Leishmania* strains from PC1 to PC4 where the variance explained per PC is about 80.35% at PC4 (Fig. 4a).

In PC1 versus PC2, a good separation of the *L. infantum* strains IMT151 and IMT373 located in the second quadrant from the remaining groups can be observed apart from a small overlap of the datasets (Fig. 4a). The latter can be explained by a similarity reflected in both datasets considered as two strains of the same species originating from the same region (Portugal). In addition, the same zymodeme MON-1 is known to be genetically very homogeneous. On the other hand, a separation between both datasets occurs in PC2 versus PC3 and even in higher principal components such as in PC2 versus PC4. A full separation from the remaining noise-containing residuals (all other datsets) can therefore be achieved. This clustering can also be observed in the dendrogram illustrated in Fig. 6.

Furthermore, for the *L. infantum* strains IMT151 and IMT373 modes at approximately 2925 cm^–1^ and 2850 cm^–1^ could be assigned to fatty acid compounds that correlate with the PC score values. These spectral contributions can also be observed in the PC loadings spectrum (Fig. 4b). The loadings spectrum represents the relationship between the original spectral data space and the new PC space and hence can be compared with the second derivative spectra, which were the inputs for the PC analyses.

In plot PC1 versus PC3 a differentiation of the *L. tarentolae* strain compared with the other strains were observed (Fig. 4a). These results correspond with other phylogenetic studies based on the cytochrome b and DNA polymerase alpha gene sequences in which *L. tarentolae* is found in a separate cluster [50–52]. According to the known classification of the genus *Leishmania L. tarentolae* is designated to the distinct subgenus *Sauroleishmania*, whereas the remaining studied species belong to the subgenus *Leishmania.* A separation of the clusters *L. donovani* and *L. tropica* was also observed. These two species belong to different species complexes within the genus *Leishmania* and the phylogenetic relationship has been proven among others by MLEE [17, 53] and the sequence analysis of many genetic loci such as the SSU (small subunit) and ITS (Internal Transcribed Spacer) region of the ribosomal DNA [54], cytochrome b [50], DNA polymerase alpha and RNA polymerase large subunit [51,52], heat shock protein 70 (*hsp70*) [55], and further ones addressed in refs. [15, 56–60]. A separation of the species *L. donovani* and *L. tropica* could be also observed and was evidenced by Mouri et al., who conducted cluster analysis based on mass spectrometrical datasets originating from sample pellets of promastigote cultures [61]. In the PC1-PC4 plots one can see that the clusters of *L. donovani* and *L. tropica* are mainly located in the domain with positive scores with respect to PC1 versus PC2 (and for *L. tarentolae* at PC1 versus PC3), whereas the scores of the remaining strains lie in the negative codomain. This arrangement of clustered datasets can also be observed in the dendrogram (Fig. 6).

This multivariate approach also entailed the analysis of the PCA loadings spectra, the latter of which can be considered for elucidating what type of vibrational mode contributes to which PC. The strain of *L. infantum* is separated in the scatter plots in Fig. 4a at PC1 versus PC2, in the second quadrant from the remaining strains where negative score values in PC1 and positive score values in PC2 can be found (Fig. 4b). Modes with a negative intensity value in PC1 and a positive intensity value in PC2 of the loadings spectrum are responsible for the separation of the *L. infantum* strains from the remaining ones.

The bands at 2921cm^–1^ and 2915 cm^–1^ are crucial for the separation capability between the species *L. donovani* and *L. tropica*. The modes at around 2915 cm^–1^ and 1660 cm^–1^ (fatty acid region) are crucial for the negative correlation of the strains *L. tarentolae*, *L. donovani*, and *L. tropica* in PC1 and therefore are responsible for the separation from the remaining strains.

The modes that are about 2920 cm^–1^ and 2850 cm^–1^ from the fatty acid regions could be successfully implemented as indicator bands for the discrimination of different stages of malaria parasites by taking loadings spectra into account [62]. The strain of *L. tarentolae* shows spectral differences with respect to amide I and amide II modes at around 1660 cm^–1^ and 1550 cm^–1^ (Fig. 2).

**Fig. 5:**
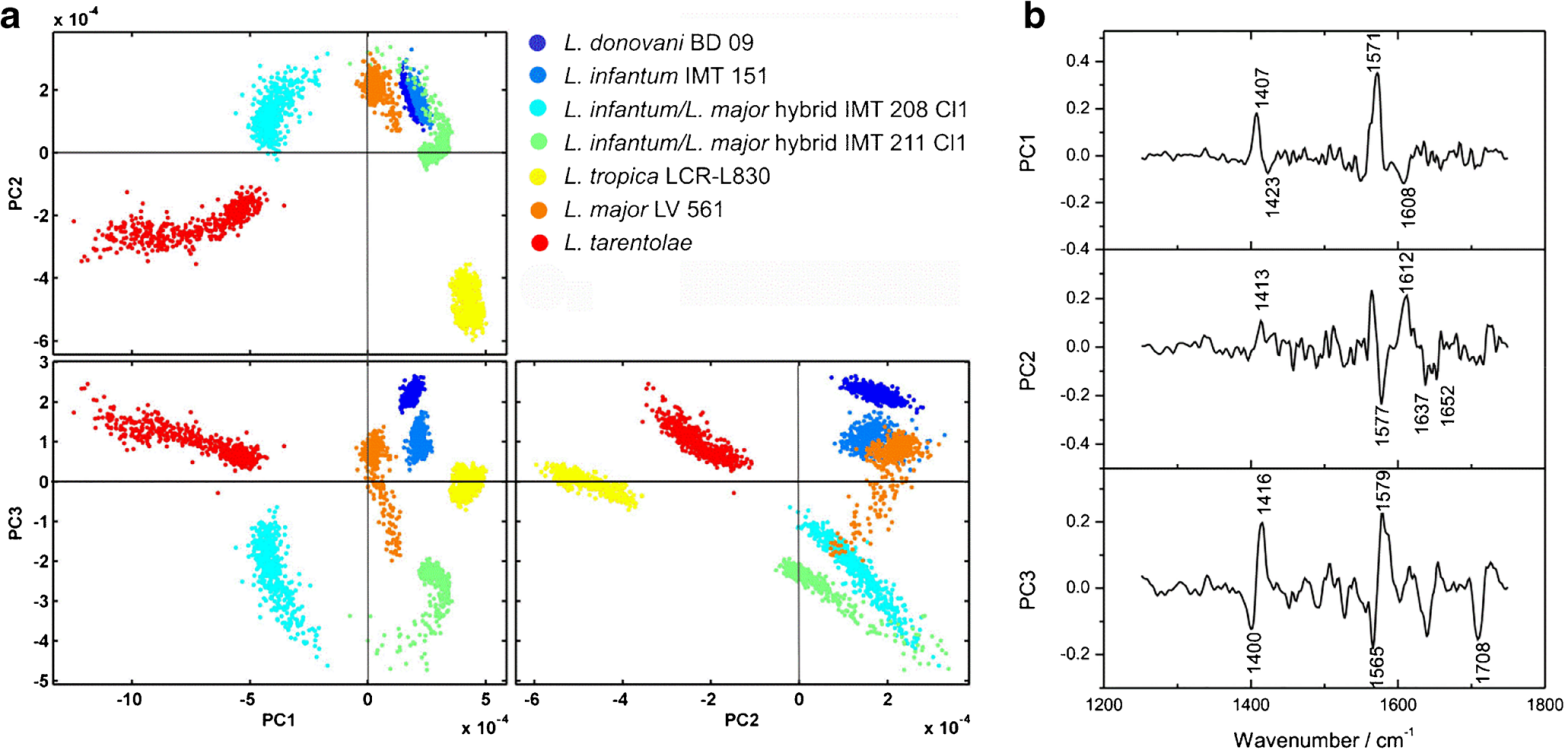
**(a)** Scores diagram of the PCA on parasite DNA films from PC1 to PC3. Analysis was performed applying 2^nd^ derivatives in the spectral windows 1750–1450 cm^–1^ and 1450–1250 cm^–1^. Datasets comprised 480 spectra for the strains *L. donovani* BD 09*, L. infantum* IMT 151, *L. infantum/L. major* hybrids IMT 208 Cl1 and IMT 211, *L. tropica* LCR-L830*, L. major* LV 561, and *L. tarentolae,* respectively. **(b)** Corresponding loadings spectra

For the analyses on DNA films the strains *L. donovani* BD09, *L. infantum* IMT151, *L. infantum/ L. major* hybrid IMT208 Cl1, *L. infantum/L. major* hybrid IMT211 Cl1, *L. tropica* LCR-L830, *L. major* LV561, and *L. tarentolae* were considered (Fig. 5a). Differentiation and classification of the datasets from DNA isolates of all strains was successful in PC1 versus PC3.

The PCA was conducted in the wavenumber regions 1750–1450 cm^–1^ (B) and 1450–1250 cm^–1^ (C) (Table 2). For PC1 a variance of approximately 48.09% has been documented, together with a total variance of 86.80% (Table S1, ESM). The scatter plots of the scores in PC1 versus PC3 display well-separated clusters, where a complete differentiation can be achieved at a variance of 75.51%. In the second quadrant of PC1 versus PC2, a cluster originating from the hybrid strain IMT208 Cl1 can be found. The third quadrant comprises the clustering of the *L. tarentolae* scores, which is separated from the remaining strains in PC1 versus PC3, and PC2 versus PC3 in the second quadrant. Along PC1 the clusters of *L. tarentolae* and *L. infantum/ L. major* IMT208 Cl1 are negatively correlated towards the other strains (cf. PC1 versus PC2, and PC1 versus PC3). These differences between the *L. tarentolae* strain and strain IMT208 Cl1 are caused by the modes at 1608 cm^–1^ (NH_2_ and C=N, adenine) and at about 1548 cm^–1^ and 1423 cm^–1^ (NH and CH base residues). A differentiation of the strains of *L. donovani*, *L. infantum*, *L. major*, and the hybrid IMT211 Cl1 is achieved in PC1 versus PC3.

Similar to the above discussed loadings spectra of the *Leishmania* strains and the loadings data for the DNA (Fig. 5b), information is provided on modes that contribute to their differentiation and separation. These bands are in the spectral window between 1750 cm^–1^ and 1250 cm^–1^.

The hybrid strain IMT208 Cl1 is separated in PC1 versus PC2 (second quadrant) from the remaining ones (Fig. 5a), which can be explained by spectral differences of the mode at 1610 cm^–1^ (cytosine ring). Together with the hybrid strain IMT211 Cl1 a negative correlation can be observed along PC3, which may be due to the spectral discrepancies at approximately 1577 cm^–1^ (thymine ring), 1637 cm^–1^ (adsorbed H_2_O molecules), and at 1652 cm^–1^ (cytosine ring). Similarity among hybrid strains can be observed in the fourth quadrant (PC2 versus PC3), which is due to the mode at 1565 cm^–1^ (NH_2_ and C=N, adenine). The spectral feature at about 1580 cm^–1^ (cytosine ring) is responsible for the differentiation of *L. tarentolae*, the scores values of which are clustered in PC1 versus PC3 and PC2 versus PC3 (second quadrant), respectively. The separation of the *L. tropica* strain occurs in the scatter plot PC1 versus PC2 (fourth quadrant), for which the mode at 1575 cm^–1^ is responsible (thymine ring).

### Differentiation of intact *Leishmania* parasites and their DNA by HCA

To perform differentiation on the variance-weighted datasets the distance-based HCA was implemented for building up dendrograms, the latter of which can be compared with the current taxonomy of *Leishmania* [1, 21, 63, 64] .

**Fig. 6:**
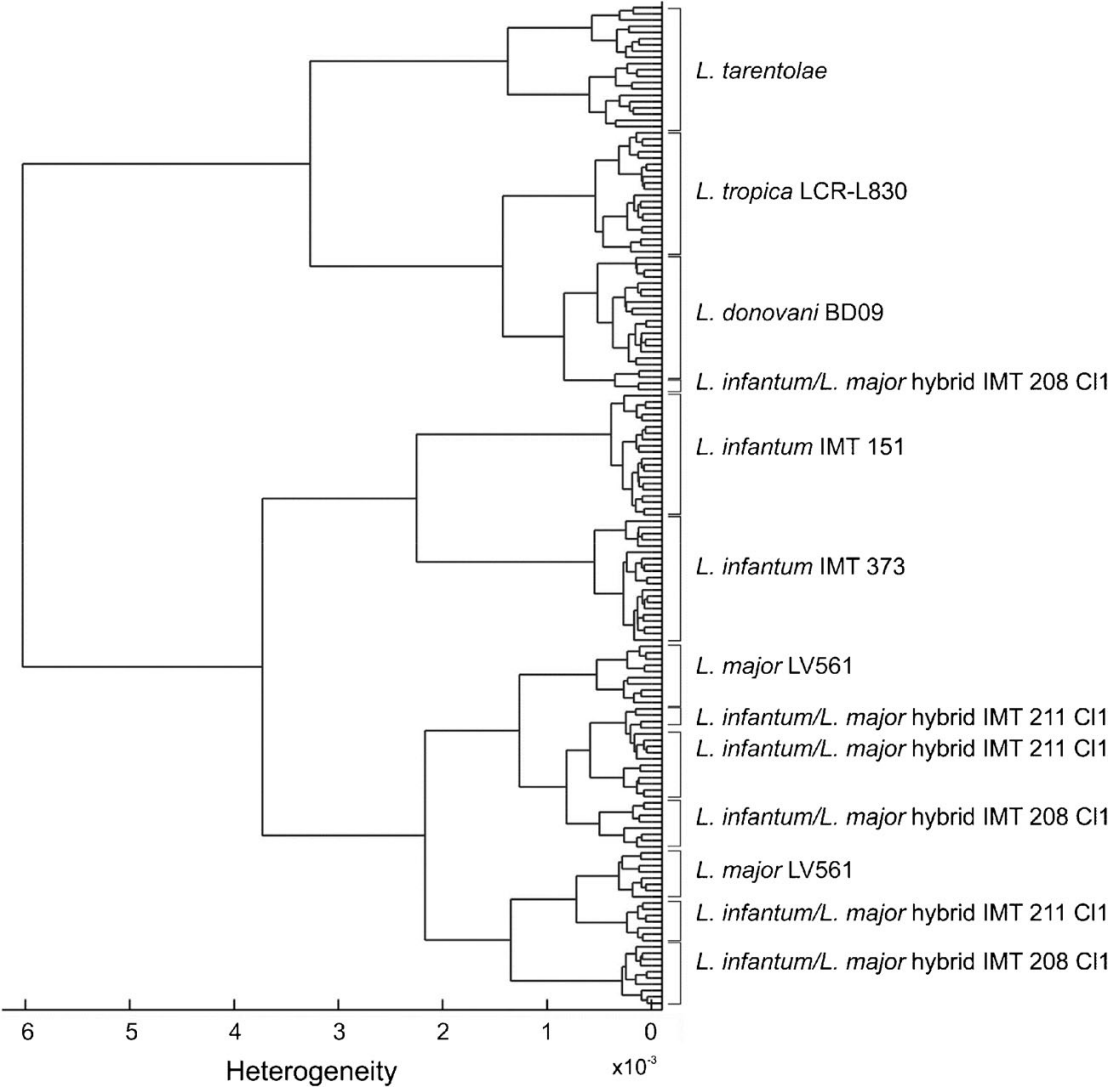
Dendrogram (phenetic tree) of the HCA on intact parasite films of eight strains investigated in the spectral windows 3000–2700 cm^–1^ and 1800–1500 cm^–1^ considering, respectively, 20 score(s) values for the strains *L. tarentolae, L. tropica* LRC-L830*, L.donovani* BD09*, L. infantum/L. major* hybrids IMT208 Cl1 and IMT211 Cl1, *L. infantum* IMT 151, *L. infantum* IMT 373, and *L. major* LV561*.* Classification of a phenetic relationship was determined using the Euclidean distance and Ward’s clustering algorithm

Figure 6 displays HCA results of the studied intact *Leishmania* parasites that are hierarchically clustered in a dendrogram structure where 20 scores values for each studied strain were considered. *Leishmania tarentolae* is the most distant species and is considered a member of distinct subgenus, whereas *L. tropica* and *L. donovani* (as well as *L. infantum* and *L. major*) belong to the subgenus *Leishmania*. The HCA illustrates that *L. tarentolae*, *L. donovani*, and *L. tropica* are clearly delimitable forming separate species-specific clusters (based on a single strain). This also applies to *L. infantum*, but here, additionally, also strain-specific sub-clusters can be recognized, as two strains of this species were included. The sub-clusters of the two *L. infantum* strains are separated at H~2.25 × 10^–3^. There is no consistent cluster for each of the hybrid strains (IMT211 Cl1 and IMT 208 Cl1) as well as for *L. major* LV561. Of notice is that the species complexes *L. major* and *L. tropica* are not closer related to each other than to the *L. donovani* complex as should be expected from DNA sequence-based classification of the *Leishmania* genus. This is due to the different band positions in the amide I and amide II region, the latter of which are mutally closer to each other for *L. tropica* and *L. donovani*. Also the distant phylogenetic relationship of *L. tarentolae* as member of a distinct subgenus is not reflected in the present dendrogram.

In the present study, HCA on *L. major*, which is the main representative of the *L. major* species complex, also shows that the score values (branches) are scattered into two different sub-clusters. These sub-clusters are intermingled with the two *L. infantum/L. major* hybrid strains. This is due to spectral differences in the polysaccharide-, phosphodiester bond-, and fatty acid region.

At a heterogeneity level (H) of about 6 × 10^–3^ the strains are clustered into two main groups: the first group includes *L. tarentolae*, *L. tropica* LCR-L830, and *L. donovani* BD09 as well as the minor part of the scores of the hybrid strain IMT208 Cl1. Within this main group, a sub-cluster of *L. tarentolae* is further separated at H~3.3 × 10^–3^ from the sub-cluster that comprises *L. tropica*, *L. donovani*, and part of the hybrid IMT 208 scores. The second main group comprises *L. infantum* IMT151, *L. infantum* IMT373, *L. major* LV561, and the hybrid strains IMT208 Cl1 (majority of scores) and IMT211 Cl1. The HCA outputs basically delineate two different main clusters, which do not agree with the current phylogeny based on genetic markers, e.g., of *L. infantum* and *L. donovani* that should be closely related as members of the same species complex and also the positions of *L. tropica* and *L. tarentolae*. The dendrogram rather shows the potential of FTIR for species discrimation or typing rather than a phylogenetic classification.

Concerning other spectrometric approaches, several MALDI-TOF mass spectrometric studies have been applied for *Leishmania* species discrimination [61, 65]. Culha et al. found species-specific spectra for the four investigated reference strains of *L. tropica*, *L. major*, *L. infantum*, and *L. donovani*, which subsequently were successfully used to identify cultured clinical isolates from patients [65]. Mouri et al. [61] were able to generate species-specific spectra from cultured *Leishmania* isolates, including also *L. donovani*, *L. infantum*, *L. major*, and *L. tropica*. The obtained dendrogram was consistent with the classification based on reference methods as MLST or sequence analysis of the *hsp*70 gene.

So far, there is only a single published FTIR-based approach (Aguiar et al.) that addresses, in combination with multivariate statistic tools, the discriminatory power and classification capability of this method tested for three *Leishmania* strains representing three species, namely *L. amazonensis*, *L. chagasi* (synonym of *L. infantum*), and *L. major*, but only by implementing HCA [32].

In PCA of intact *Leishmania* parasites, a small overlap between *L. infantum* IMT151 and IMT373, both located in the second quadrant, was observed (Fig. 4a). This can be explained by a similarity that is reflected in both datasets as both strains are of the same species also belonging to the genetically very uniform zymodeme MON-1. A separation between both datasets occurs in PC2 versus PC3. However, HCA on these strains clearly shows that they are strictly separated forming two distinct subclusters within the *L. infantum* cluster (Fig. 6). Very promising in terms of species and strain typing is that both *L. infantum* strains are mainly located in sister-groups within a species-specific cluster expected from taxonomic considerations.

Interestingly, *L. major* LV561 and both hybrid strains do not form any clearly separable strain-specific sub-clusters; instead they show a mixed topology of two sub-clusters, each including *L. major* LV561, and the two *L. infantum/L.major* hybrids IMT 208 Cl1 and IMT 211 Cl1. This suggests a higher similarity of the hybrids to the *L. major* parent strain. This was also noticed in the PC scatter plots; none of the six displayed plots showed a clear separation of *L. major* LV561 and both hybrid strains. In a previous study it was shown that polyploidy observed in experimental *L. infantum/L. major* hybrid progenies displayed distinct tropisms, in terms of clinical forms, depending on the parental origin of extra chromosomes [66]. Only parts of the *L. infantum/L. major* hybrid IMT208 are located close to *L. donovani*. However, not in the *L. infantum* cluster, which can be explained by emerging spectral outliers, which concerns two datasets, as a split of the IMT208 scores values (Fig. 6). This may have been caused by the high spectral variability in the spectral region between 3550 cm^–1^ and 3250 cm^–1^, which is nearly in the same order of magnitude as for the *L. donovani* datasets (compare standard deviations in this region for both strains datasets in Fig. 2).

Interesting is the position and behavior of the remaining hybrids – intermingled with *L. major*. The *L. infantum/ L. major* hybrids display two subclusters here, together with a split of *L. major* into two subclusters. This is due to the following reasons: Within the spectra of the *L. infantum/L. major* hybrid IMT208 spectral differences can be observed; for instance, in the polysaccharide region at about 1174 cm^–1^, and phosphodiester bond region at about 1085 cm^–1^, as well as in the fatty acid region at about 3078 cm^–1^. This observation can also be made for the datsets of the *L. infantum*/*L. major* hybrid IMT 211, which display a spectral variability at about 1073 cm^–1^ and at about 3298 cm^–1^. The data of *L. major* LV561 only mutually differ at about 3298 cm^–1^.

**Fig. 7:**
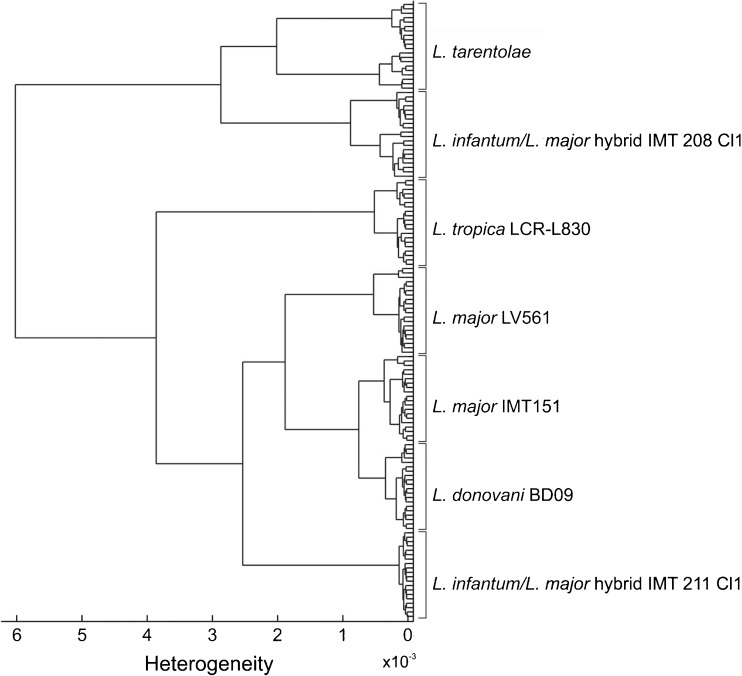
Dendrogram of the HCA on parasite DNA films investigated in the spectral windows 1750–1250 cm^–1^ considering, respectively, 20 score(s) values for the seven strains *L. tarentolae, L. infantum/L. major* hybrid IMT208 Cl1, *L. tropica* LCR-L830*, L. major* LV561*, L. donovani* BD09, *L. infantum* IMT151, and *L. infantum/L. major* hybrid IMT 211 Cl1. The phenetic tree was determined using Euclidean distance and Ward’s clustering algorithm

For the HCA on DNA films in the 1750–1250 cm^–1^ spectral range, 20 score(s) values for each of the seven studied strains have been selected (Fig. 7). It is striking that the dendrogram comprises uniform strain-specific clusters and no outliers can be found as in Fig. 6. In the dendrogram two groups are separated at H of about 6 × 10^–3^. The first group entails clustered datasets of *L. tarentolae* and of the hybrid IMT208 Cl1, the latter of which branch out at an H~2.9 × 10^–3^. The remaining strains can be found in the second clustered group. *L. tropica* LRC-L830 with its branching off at the second highest H value of about 3.9 × 10^–3^ reflects a relatively distinct taxonomic entity.

One further branch can be found at H~2.5 × 10^–3^, which leads to the sub-cluster of the hybrid IMT211 Cl1, and to the sub-cluster that comprises *L. major* LV561; *L. infantum* IMT151 and *L. donovani* BD09. *L. major* LV561 is separated at H~1.9 × 10^–3^ from the subgroup of *L. infantum* IMT151 and *L. donovani* BD09. The separation of *L. infantum* from *L. donovani* was observed at H~1.8 × 10^–3^. The dendrogram shows the highest similarity between *L. infantum* and *L. donovani* at a value of H~1.8 × 10^–3^, which is in agreement with the current taxonomy with these two species belonging to the *L. donovani* species complex.

For both parasite films and those of the DNAs, the MIR signatures are mutually very similar. However, there is a slight difference of the molecular profiles of parasites and DNAs of the strain since it includes (or considers) different amounts of molecular constituents (compare Tables 3 and 4) that are responsible for the respective PCA and HCA outputs.

DNA film spectra do not exhibit any large variations in their PCA- and HCA-based clustering compared with the intact parasite film datasets, which can be explained by a lesser complex biochemical composition. The (spectral) variability of DNA data within one strain is lower compared with the parasites. This can be explained by a higher homogeneity within sample preparation of DNA films, as the multiple drop-casted amount comprised nearly the same molecules. In contrast, the parasite films entailed a larger spectral variety and complexity, which is due to the additional modes of different molecules, i.e., modes that refer to fatty acid residues, proteins, and peptides. In addition, the sample films comprised microstructures, likely disordered by the whole parasitic organisms, may have also caused spectral variablities. Indeed, slight spectral differences could also be observed and traced both univariately and multivariately, between bacterial strains and some of their related PCR products [28].

The pilot study illustrates here that PCA scores were successfully implemented for cluster analyses, PCA functioned as a complementary multivariate analysis tool, whereas HCA enabled the disposition of spectral datasets of parasite films in a hierarchical order to phenetic dendrograms.

FTIR complementary tools, such as Raman spectroscopy, may also provide further insight into the biochemical composition of parasites and their DNAs by vibrational fingerprints. As far as we are aware, there are no publications on *Leishmania* parasites. This methodology was already applied for other parasites, e.g., for diagnosis of Malaria and Toxoplasmosis [67–69].

## Conclusions

This work comprises a pilot study on the molecular composition of five *Leishmania* species*, L. infantum/L. major hybrids* and their corresponding DNA from the Old World. Each was successfully investigated by FTIR micro-spectroscopy. Chemical univariate analysis has provided insights into molecular structure and composition both for whole *Leishmania* parasites and their extracted DNA. The parasites could be discriminated by spectral differences because of the polymorphism of polysaccharides, as well as different contributions from fatty acids such as phospholipids, nucleic acids, and proteins (amides region). Considering the DNA datasets, discrimination capability was achieved by spectral differences of base residues, i.e., contributions from thymine and cytosine, and from the phosphate-deoxyribose backbone.

The *Leishmania* species differentiation has been illustrated in two different spectral windows, addressing a systematic approach underpinned by multivariate statistics tools such as PCA and HCA.

PCA allowed a distinct identification and discrimination by unique MIR spectral fingerprints of *Leishmania* and their DNAs at the respective wavenumber windows, enabling successful segregation between information-rich and -poor spectral components. Considering the PCA and HCA results of the DNA, a better differentiation could be achieved than for the parasites. Hence, DNA may be a more reliable candidate for discrimination due to the possible elimination of the environmentally sourced changes on the complex biochemical content of the cells.

PCA results have shown that the clustered spectral datasets in the scores diagrams strongly correlate with the clusterings in the HCA. However, a phenetic classification was only feasible in combination with HCA.

At this time, the present results indicate the suitability of FTIR for typing/identification rather than for phylogenetic classification purposes. Further *Leishmania* species and strains must be investigated in order to elucidate species-specific signatures that will allow correct identification. Consequently it is crucial to strive for further investigations related to intra-species variability and species-specific clustering.

Another relevant limitation of the FTIR method is the need of isolation of *Leishmania* parasites from biological samples and culturing, making this process more time-consuming in the steps that precede this methdology FTIR. An adaptation for the direct use of clinical material should be further tested.

As vibrational spectroscopic characterization of *Leishmania* species and their molecular components (DNA) with respect to their biochemical compositions by means of FTIR comprises a relatively new approach, FTIR data of *Leishmania* are scarce. This evokes the impetus for further systematic evaluation based on a balanced and representative sample set and consideration of other parasitic species [32] up to the single-cell level [70].

## Electronic supplementary material

ESM 1 (PDF 337 kb)
