# Supplementary material for: A pilot study on fingerprinting Leishmania species from the Old World using Fourier transform infrared spectroscopy
Source: Anal Bioanal Chem. 2017 Oct 28;409(29):6907–23. doi: 10.1007/s00216-017-0655-5 (PMC5670197; doi:10.1007/s00216-017-0655-5)
Supplement: Supplementary file 1 — (PDF 337 kb) [file 216_2017_655_MOESM1_ESM.pdf]

Analytical and Bioanalytical Chemistry

Electronic Supplementary Material

A pilot study on fingerprinting *Leishmania* species from the Old World using Fourier transform infrared spectroscopy

Andrea Hornemann, Denise Sinning, Sofia Cortes, Lenea Campino, Peggy Emmer, Katrin Kuhls, Gerhard Ulm, Marcus Frohme, Burkhard Beckhoff

Further details on Material and Methods– Isolation of Leishmania-DNA

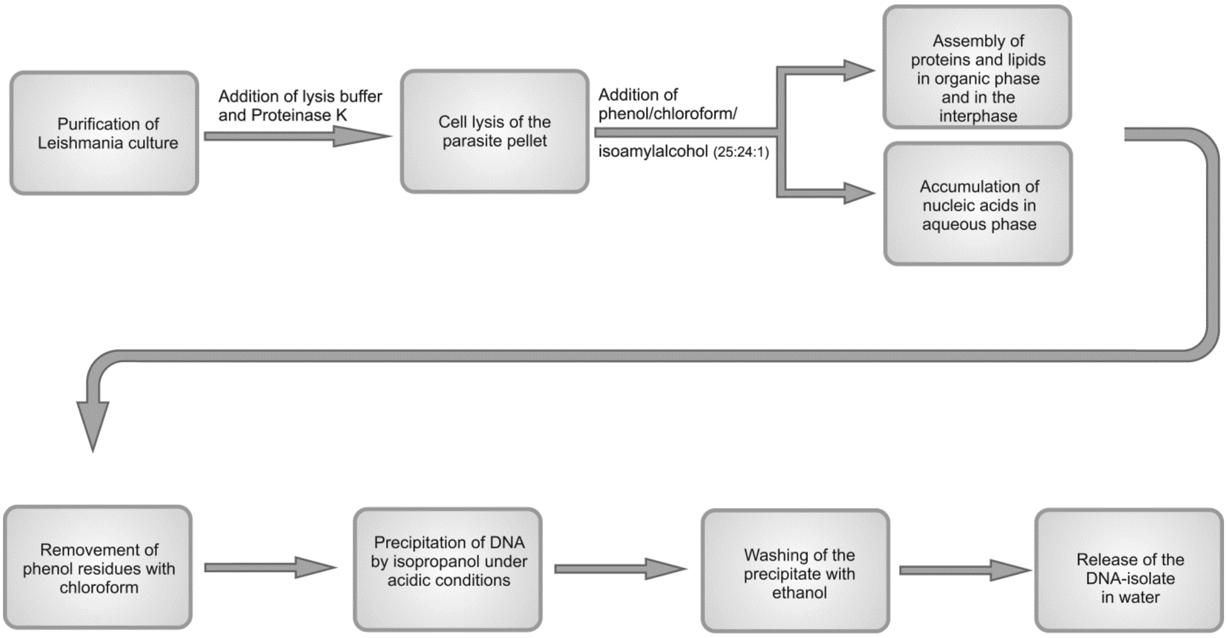

Fig. S1 Workflow of the preparation steps of the phenol/chloroform/isoamylalkohol-extraction for the respective DNA of *Leishmania* strains

Further details on Results and Discussion – Univariate studies / Chemical analyses on parasite films and DNAs

For studying the consistency and reproducibility of *Leishmania* film data, their normal distribution was studied by the construction of boxplots. The boxplots provide an overview of the diverse robust measures of dispersion and distribution of datasets (Fig. S2, S3). Further, the position, concentration and variation between several datasets can be envisaged. Normalized arithmetic means were chosen for the boxplots by taking 2 repetitive sample measurements (in triplicate) for the five strains *L. infantum* IMT 151, *L. infantum/L. major* hybrids IMT 208 Cl1

and IMT 211 Cl1, *L. donovani* BD09, and *L. tarentolae* into consideration, and for the remaining samples 1 repition was considered (in duplicate).

Fig. S2 displays upper / lower whiskers (├ / ┤) that highlight the minimum and maximum values of the absorbance between 0 and 1 in all boxplots which is due to the normalized datasets.

The boxes describe the area in which 50% of the datasets lie. For all strains the datasets of the respective film preparations display a good reproducibility as all boxes lie more or less at the same absorbance level, and inter-quartile distances (length of the box) are similar among each other within the sample preparations, apart from some deviations, which can be observed for BD12 (*L. donovani*) for instance. Possible reasons for these spectral discrepancies can be attributed to different variations of the protein / lipid content. The latter can be detected in different strains but may also differ for the respective life cycle of *Leishmania* parasites [1].

The boxplots for the experiments of the strains *L. infantum/L. major* hybrid IMT 208 Cl1 and *L. donovani* BD09 confirm the uniformity of the distribution of spectral data in one strain for three single preparations. Additionally, uniformity is underpinned due to the same data preprocessing procedures (i.e. baseline correction, vector-normalization, mean-centering, 2nd derivatives and Savitzky Golay smoothing).

For the *L. infantum/L. major* hybrid IMT 208 Cl1, the regions in which 50% of all data lie are located at very similar absorbance levels ranging from about 0.02 to 0.30 (cf. preparations 1 and 3), and for preparation 2 the absorbance is in the range between 0.08 and 0.22. The median (—) is located at absorbance values between 0.12 and 0.15, and the arithmetic mean value at ca. 0.19 – 0.20. For *L. donovani* BD09 the median is located between 0.1 and 0.14 and the arithmetic mean value is between 0.14 and 0.15 for the three preparations. The inter-quartile distance is more stable for the three preparations as for the *L. infantum/L. major* hybrid IMT 208 Cl, as 50% of the data have absorbance values between 0.02 and 0.2.

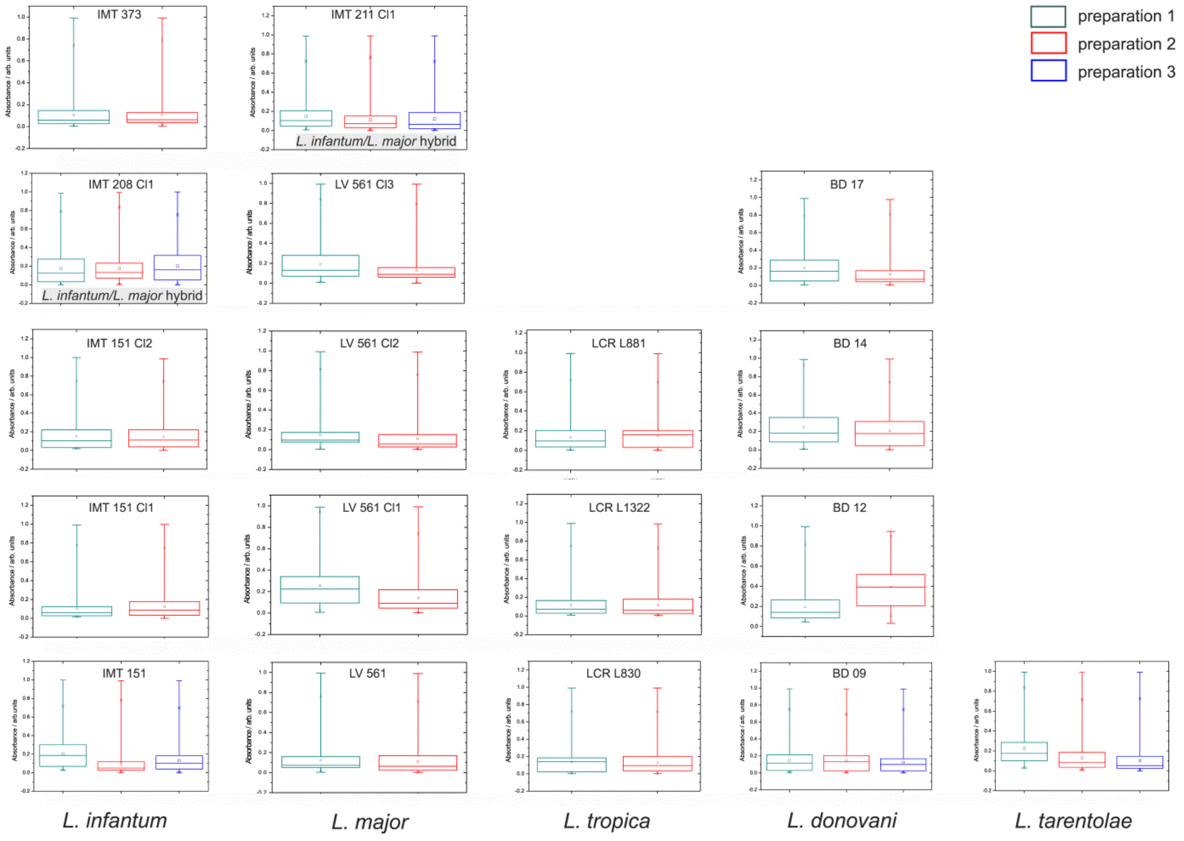

Fig. S2 Boxplots of IR datasets (3900 cm^{-1} - 900 cm^{-1}) acquired from repetitive preparations (preparations 1 - 3: □, □, □) of *L. infantum, L. major, L.infantum/L. major hybrids, L. tropica, L. donovani, and L. tarentolae* parasite films. Crosses (×) illustrate values in between which 1% und 99% of the data lie, squares in boxes (□) display the arithmetic mean value and cross-lines the median (—)

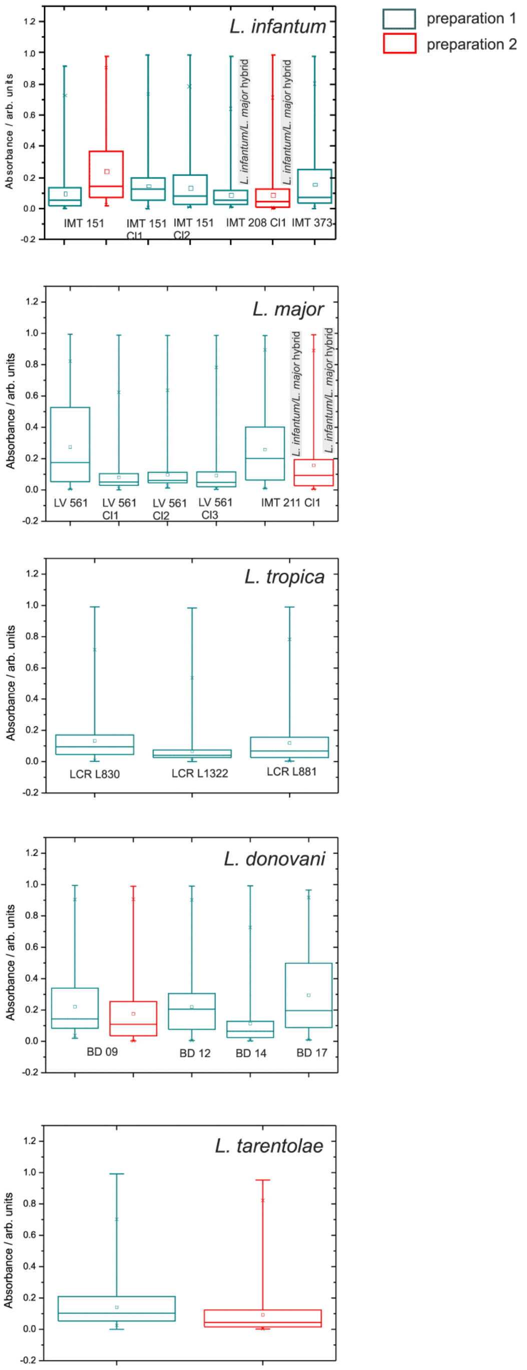

Fig. S3 Boxplots IR datasets (3900 cm^{-1} - 900 cm^{-1}) acquired from repetitive preparations (preparations 1 - 2: □, □) of *L. infantum, L. major, L.infantum/L. major hybrids, L. tropica, L. donovani, and L. tarentolae* DNA films. Crosses (×) illustrate values in between which 1% und 99% of the data lie, squares in boxes (□) display the arithmetic mean value and cross-lines the median (—)

Details on Results and Discussion – PCA analyses on parasite and DNA sample films

Table S1 Explained variances of the conducted PC analyses on parasite and DNA films at the respective wavenumber windows and their combinations

Wavenumber window	PC1-explained variance / %	Total variance / %
Intact parasite films:		
complete spectrum	20.83	58.21
W1	12.16	35.83
W2	46.16	85.78
W3	22.71	54.36
W4	22.53	58.68
W1, W2, W3 and W4	39.78	73.52
W2, W3 and W4	24.32	64.71
W1 and W2	46.52	83.87
W2 and W3	36.04	79.25
W2 and W4	75.27	88.91
W3 and W4	18.23	50.29
DNA films:		
complete spectrum	17.07	56.52
A	6.06	26.16
B	49.73	88.31
C	53.81	84.43
D	17.77	51.40
A, B, C and D	46.52	81.12
B and C	48.09	86.80

References

1. Aguiar JC, Mittmann J, Ferreira I, Ferreira-Strixino J, Raniero L. Differentiation of Leishmania species by FT-IR spectroscopy. Spectrochim Acta Part A Mol Biomol Spectrosc. Elsevier B.V.; 2015;142:80–5.
